# Advances in Applying Computer-Aided Drug Design for Neurodegenerative Diseases

**DOI:** 10.3390/ijms22094688

**Published:** 2021-04-28

**Authors:** Mootaz M. Salman, Zaid Al-Obaidi, Philip Kitchen, Andrea Loreto, Roslyn M. Bill, Richard Wade-Martins

**Affiliations:** 1 Department of Physiology, Anatomy and Genetics, University of Oxford, Parks Road, Oxford OX1 3QX, UK; andrea.loreto@dpag.ox.ac.uk; 2 Oxford Parkinson’s Disease Centre, University of Oxford, South Parks Road, Oxford OX1 3QX, UK; 3 Department of Pharmaceutical Chemistry, College of Pharmacy, University of Alkafeel, Najaf 54001, Iraq; Zaid.alobaidi@alkafeel.edu.iq; 4 Department of Chemistry and Biochemistry, College of Medicine, University of Kerbala, Karbala 56001, Iraq; 5 School of Biosciences, College of Health and Life Sciences, Aston University, Aston Triangle, Birmingham B4 7ET, UK; p.kitchen1@aston.ac.uk (P.K.); r.m.bill@aston.ac.uk (R.M.B.); 6 John Van Geest Centre for Brain Repair, University of Cambridge, Cambridge CB2 0PY, UK

**Keywords:** neurodegeneration, drug discovery, CADD, dementia, brain diseases, CNS disorders, Alzheimer’s disease, Parkinson’s disease, amyotrophic lateral sclerosis, Huntington’s disease

## Abstract

Neurodegenerative diseases (NDs) including Alzheimer’s disease, Parkinson’s disease, amyotrophic lateral sclerosis, and Huntington’s disease are incurable and affect millions of people worldwide. The development of treatments for this unmet clinical need is a major global research challenge. Computer-aided drug design (CADD) methods minimize the huge number of ligands that could be screened in biological assays, reducing the cost, time, and effort required to develop new drugs. In this review, we provide an introduction to CADD and examine the progress in applying CADD and other molecular docking studies to NDs. We provide an updated overview of potential therapeutic targets for various NDs and discuss some of the advantages and disadvantages of these tools.

## 1. Introduction

Neurodegenerative diseases (NDs) are incurable and debilitating conditions that result in progressive degeneration and/or death of nerve cells in the central nervous system (CNS) [1,2,3]. Dementia rates are alarmingly on the rise worldwide. There are over 50 million people worldwide living with dementia in 2020, with nearly 60% living in low- and middle-income countries [4]. This number will almost double every 20 years, reaching 82 million in 2030 and 152 million in 2050 [4]. The number of people with dementia in the UK is predicted to be around 1.14 million by 2025 and 2.1 million by 2051, an increase of 40% over the next 5 years and 157% over the next 31 years [5].

The UK Prime Minister’s Challenge on Dementia was launched in 2015 to identify strategies to tackle dementia by 2025 [6]. Current therapies for NDs treat symptoms, not the underlying pathological changes. There is a clear and unmet clinical need to develop new therapies based on understanding the molecular pathologies. One of the most promising approaches is to develop novel therapeutics using computer-aided drug design (CADD) [7,8].

In this review, we provide an introduction to CADD and different approaches involved in this technique. We provide a list of over 200 pieces of CADD software using a citation-based scoring system (Supplementary Table S1), with the 30 most commonly used software products listed in Table 1. We examine the progress in applying CADD and other molecular docking studies to NDs, provide an updated overview of potential therapeutic targets for various NDs, and discuss some of the advantages and disadvantages of these tools.

## 2. Computer-Aided Drug Design

**Figure 1:**
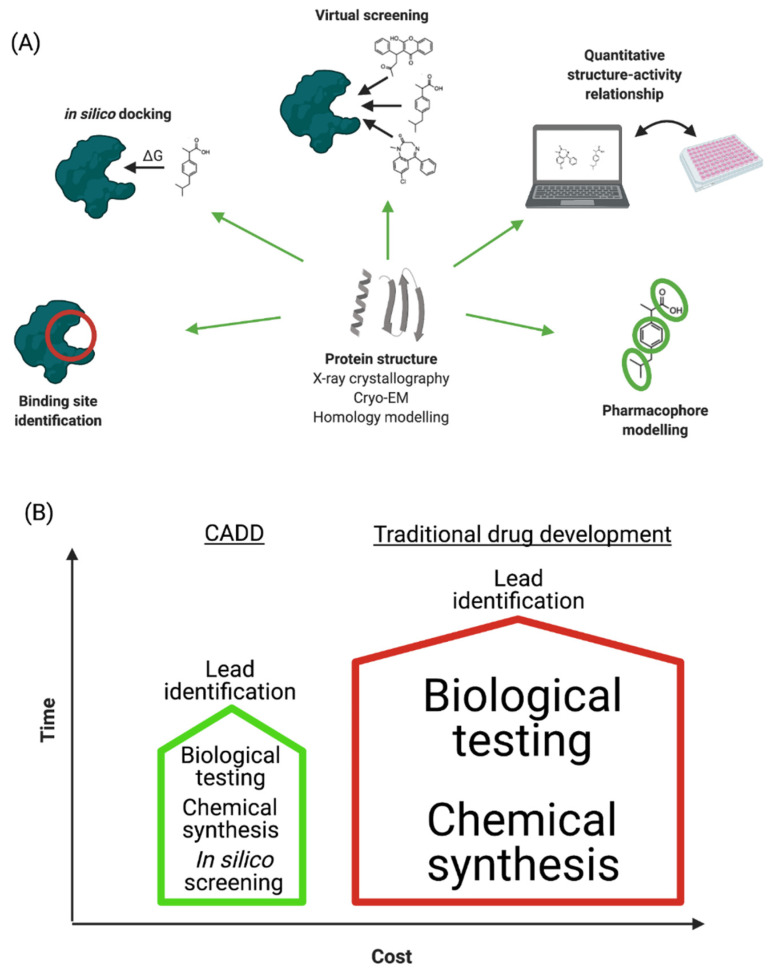
(**A**) Schematic representation of CADD process. (**B**) Comparison of traditional and computer-aided drug development in terms of time and cost investments.

“Computer-aided drug design” (CADD) refers to the application of computational modelling approaches to drug discovery. Drug discovery is an expensive and time-consuming process with the average approved drug requiring 10 to 15 years to develop with an estimated cost of 0.8–2 billion USD [9]. Many licensed drugs, such as captopril, dorzolamide, oseltamivir, aliskiren, and nolatrexed, were all optimized using CADD [10], and a large number of publications describe the successful design and discovery of leads/drugs using CADD [11,12,13]. The major steps involved in CADD are summarized in Figure 1A and discussed in the following sections. The main goal of CADD is to reduce these timescales and costs without affecting quality (Figure 1B) [14]. Importantly, CADD can be used in most stages of drug development: from target identification to target validation, from lead discovery to optimization, and in preclinical studies. It is therefore estimated that CADD could reduce the cost of drug development by up to 50% [15,16].

### 2.1. Drug Target Selection

Drug target selection is the first step of structure-based drug design. This involves identifying and determining the structures of the relevant proteins [17]. Understanding and characterization of the molecular biology of the targeted disease are therefore necessary before the initiation of any active compound search process.

### 2.2. Determination of the Protein Structure

An in-depth understanding of biological processes is still often hampered by a lack of detailed knowledge of protein structures [18]. The determination of the structure of the target protein is a prerequisite for CADD [19]. Structural elucidation of the target protein can be performed by experimental tools including, but not limited to, nuclear magnetic resonance (NMR) spectroscopy, Cryo-EM, and X-ray crystallography [20,21].

### 2.3. Homology Modelling

Despite the current revolution in structural studies, in particular the recent developments in cryo-EM, the detailed structures of a large number of proteins, and especially membrane proteins (which are over-represented amongst drug targets), have not been determined [18,22]. Homology modelling is an approach to estimate the structure of a target protein based on structural data from proteins with sequence homology to the target [23].

For instance, a homology model of human catechol-O-methyltransferase (COMT) was constructed utilizing the X-ray crystal structure of rat COMT to design anti-PD drugs by performing ligand docking, resulting in the discovery of nine putative inhibitors. Another example involves a cysteine protease from *Xanthomonas campestris* (an aerobic, Gram-negative rod-shaped bacterium known to cause black rot in crucifers by darkening the vascular tissues). The active site of this enzyme is homologous to human cathepsin B enzyme (hCB), the activity of which contributes to the reduction of the amyloid peptide by proteolytic cleavage of Aβ1-42, offering a protective role against AD [24].

### 2.4. Identification of Binding Sites

When the three-dimensional structure of the target protein is determined, the next step is the identification of potential binding sites for small molecules. This process can be conducted using various algorithms for computing and identifying binding pockets [25,26,27].

### 2.5. Molecular Dynamics Simulation

Molecular dynamics (MD) simulations are a theoretical tool to discover the configurations and dynamic behaviours of molecules, providing atomic-level insight into drug mechanisms of action [13]. MD may also help to reveal the aggregation pathway of neurotoxic protein aggregates and thus aid in the design of new inhibitors [28].

### 2.6. Molecular Docking Studies

Molecular docking is a computational procedure that predicts the lowest energy binding conformations of one molecule to a second (usually a small drug-like molecule to a protein). Accordingly, molecular docking procedures, along with their different scoring systems, are frequently utilized to predict the binding modes and affinities between chemical compounds and drug binding sites on biological macromolecules [29,30].

### 2.7. Virtual Screening

Virtual screening (VS) is the process of screening small molecule libraries in silico to identify chemical structures that may bind to a drug target [31,32,33].

### 2.8. Quantitative Structure—Activity Relationship Study

Quantitative structure—activity relationship (QSAR) methods are conducted to correlate a biological response (e.g., enzyme activity, cell viability, etc.) to the chemical properties of a set of molecules [34,35,36].

### 2.9. Pharmacophore Modelling

Pharmacophore modelling deals with finding the optimal shapes and charge distributions for binding of a small molecule to a biological macromolecule. Pharmacophore modelling is commonly implemented to rapidly specify potential lead compounds [37,38].

## 3. Neurodegenerative Diseases

**Figure 2:**
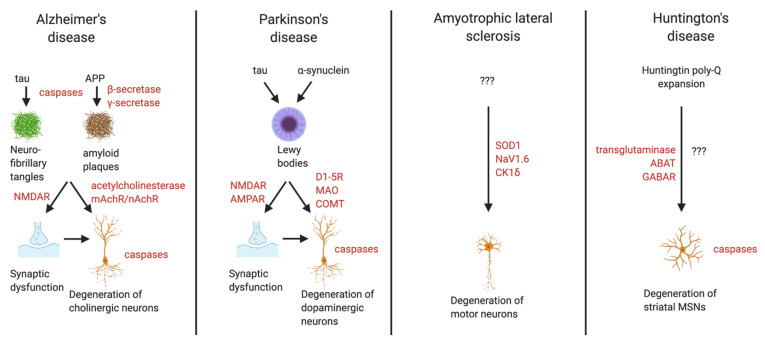
Overview of molecular mechanisms and drug targets (red text) in Alzheimer’s, Parkinson’s, ALS, and Huntington’s diseases. Figure made using Biorender.com, accessed on 28 April 2021.

NDs include Alzheimer’s disease (AD), Parkinson’s disease (PD), amyotrophic lateral sclerosis (ALS), and Huntington’s disease (HD) [39]. These diseases are diverse in their pathophysiology and effective treatments are urgently needed, but they will only be achieved with an in-depth understanding of the causes and mechanisms of each disease. These diseases and potential drug targets for each are discussed briefly below. Current molecular targets for these diseases, along with examples of drugs discovered in CADD projects, are summarised in Table 2. The molecular mechanisms of neurodegeneration and potential drug targets in these diseases are summarised in Figure 2.

### 3.1. Alzheimer’s Disease (AD)

AD is a chronic, progressive, and persistent neurodegenerative disease whose main symptoms are reduced motor and cognitive function and accelerated memory loss, resulting from the progressive loss of neurons and synapses in the cerebral cortex, ultimately leading to death [66].

The estimated number of people aged 65 years or older in the USA with AD in 2010 was 4.7 million and this number is predicted to reach 13.8 million by 2050 [67]. In 2013, the number of people in the UK with dementia was estimated at 815,827, of which 62% had AD [68]. Approximately 70% of the UK care home population suffers from dementia and more than 42,000 people below 65 years also have dementia [69].

AD is characterised by the presence of amyloid plaques, composed primarily of aggregated amyloid-β (Aβ) peptides proteolytically derived from the amyloid precursor protein (APP), and neurofibrillary tangles (NFTs) that are intracellular protein aggregates composed primarily of phosphorylated tau protein. Although amyloid deposits are thought to develop before NFTs, amyloid burden is poorly correlated with disease progression, whereas NFT burden is more strongly correlated [70]. The exact mechanisms by which NFTs and Aβ plaques lead to neurodegeneration are still poorly understood. Several genetic contributors to AD have been identified, including variants of presenilin 1 (PSEN1) and presenilin 2 (PSEN2), components of the γ-secretase complex that cleaves an APP intermediate to its amyloidogenic forms, as well as variants of APP itself. The strongest genetic risk factor not directly involved in amyloid formation is the APOE gene, encoding an apolipoprotein that is responsible for CNS cholesterol transport. Weaker genetic risk factors include a variety of genes involved in cholesterol metabolism, endocytosis, and neuroinflammation [71,72]. Recent work on the glymphatic waste clearance system suggests that reduced glymphatic function is correlated with Aβ and tau accumulation [73]. Bulk flow through the glymphatic system is elevated during sleep and mediated by the water channel protein AQP4, which is also implicated in various CNS pathologies [74,75,76]. Despite there being no single drug that has been approved to successfully target AQP4 [77], new studies suggest that modulators of sleep or AQP4 (by targeting the trafficking mechanism or membrane abundance rather than pore-blocking) could be novel targets for early intervention in AD and other protein-misfolding diseases [78,79].

#### 3.1.1. Macromolecular Targets in AD

##### Acetylcholinesterase

Acetylcholinesterase inhibitors (AChEIs) have been considered as potential drugs to treat AD and other dementias for many years, due to the degeneration and loss of cholinergic neurons associated with AD symptoms. Indeed, three of the four currently approved drugs for AD are AChEIs (donepezil, galantamine, and rivastigmine). Accordingly, acetylcholinesterase is routinely targeted in docking studies [80]. For example, utilizing molecular docking, the binding of compounds found in *Salvia miltiorrhiza* (red sage) extract, e.g., miltirone and salvianolic acid A, to acetylcholinesterase [81], and the binding of cinerin C (a molecule extracted from Prosopis cineraria pods) to acetylcholinesterase [82] have been reported.

##### Beta-Secretase and Gamma-Secretase Enzymes

Aβ formation is catalysed by β-secretase (BACE) and γ-secretase (GS) enzymes and, thus, inhibiting these enzymes could prevent Aβ plaque formation and prevent AD [83]. Molecular docking has been utilized to score putative inhibitors of GS, and the highest scoring compound was used to identify chemically similar compounds for pharmacophore mapping [84].

##### Caspases

Caspases are important mediators of apoptosis in neurons (and indeed in most cell types); their inhibition might therefore be helpful in preventing neurodegeneration-associated neuronal death in ALS, AD, PD, and HD [85,86,87,88].

Several studies employed in silico drug design and molecular docking to target caspases to treat NDs. For example, ten non-cytotoxic nitrones were assessed for their capability to arrest apoptosis and reduce the levels of active caspase-3 and oxidative stress in the HT22 neuronal cell line. Molecular docking suggested that these nitrones bound to a site near the catalytic region of caspase-3. This suggested that medicinal chemistry using these nitrones as a starting point could be considered to begin the development of novel ND therapies [89].

##### Acetylcholine (ACh) Receptors

Many studies conducted both in vitro and in vivo have demonstrated that reduced cholinergic activity is a direct cause of memory loss in AD patients [90]. Consequently, one of the potential targets in AD is the nicotinic acetylcholine receptor (nAChR). Compounds discovered using multitarget CADD studies based on nicotinic receptors were found to improve memory, cognition, and spatial capabilities in animal models [91,92].

##### N-Methyl-D-Aspartate Receptor

N-methyl-D-aspartate (NMDA) receptors transduce glutamate and glycine signals that play crucial roles in CNS development and the synaptic plasticity that is essential for memory and learning processes [93]. However, overexposure to glutamate can result in neurotransmission disturbances correlated with the NMDA receptor, which are treatable with NDMA antagonists [94,95]. The identification of conantokins, MK-801 and memantine (memantine was approved by the FDA for AD in 2004), as NMDA receptor inhibitors led to the investigation of these structures using CADD to identify new NMDA receptor inhibitors. New compounds discovered in this way could be utilized as potential AD therapeutics [47,94,96,97].

##### ROCK-I and NOX2 Enzymes

One of the possible approaches to treat neuroinflammation is the inhibition of both NADPH oxidase 2 (NOX2) and Rho kinase 1 (ROCK-I). This might be an effective way to treat some progressive neurological diseases, including AD [98]. NOX2 is the catalytic subunit of a multi-protein complex that can be activated in host defence phagocytic processes (e.g., in microglia) to govern the generation of superoxide from oxygen. ROCK-I is a significant mediator of cell migration, proliferation, and adhesion. In disease states, NOX2 integration into the NADPH oxidase complex is activated by ROCK-I via Ras associated C3 botulinum toxin substrate (Rac). Consequently, microglial cells with high ROCK-I and NOX2 lead to progressive neuronal damage in the early development of neurological disease [99]. In one study, CADD was utilized to discover new molecules with the ability to inhibit both ROCK-I and NOX2, with 18 compounds identified from a library of 5 × 10^5^. Of these 18 molecules, 7 had an inhibitory effect against both enzymes in cell-based assays [98].

### 3.2. Parkinson’s Disease (PD)

PD is the second most common neurodegenerative disorder with symptoms including tremors, muscle rigidity, and postural imbalance [100,101,102]. PD affected around 145,000 people in the UK in 2019 [103,104,105]. In the USA, the estimated number of annual PD diagnoses is 60,000 and approximately one million are affected with PD in 2020 [106,107]. PD is characterised by preferential and progressive loss of dopaminergic neurons starting in the substantia nigra pars compacta, and the presence of intracellular aggregates, known as Lewy bodies, composed primarily of the protein α-synuclein. Exactly how (or even if) Lewy bodies exert neurotoxic effects is poorly understood.

#### 3.2.1. Macromolecular Targets in PD

##### COMT (Catechol-O-Methyltransferase) Inhibitors

COMT metabolises catechols by methylation. As dopamine is one of the catechols that is reduced in the CNS during PD, COMT is considered a drug target for the management of PD. Nitrocatechol-type inhibitors (e.g., tolcapone and entacapone), bisubstrate inhibitors (e.g., thiopyridine, purine, N-methyladenine, and 6-methylpurine), and other molecules (e.g., 4-phenyl-7,8-dihydroxycoumarin) were reported as potential COMT inhibitors from structure-based drug design studies [108].

##### Dopamine Agonists

Pergolide, pramipexole, ropinirole, bromocriptine, and piribedil are currently the most commonly prescribed dopamine-receptor agonists. They are generally combined with levodopa plus dopa decarboxylase inhibitors (DDIs), especially in patients with motor dysfunctions. They can be efficient as a monotherapy during early PD (they can delay the need for the introduction of levodopa plus DDIs in newly diagnosed patients) or in combination with levodopa plus DDIs for dyskinesia and motor fluctuations [109].

There are five subtypes of dopamine receptors, D1–5 and each one has a different function. Different patients may respond differently to different dopamine receptor agonists. Hence, clinicians often change the therapeutic choice from one dopamine receptor agonist to another in order to achieve better control of PD symptoms and avoid specific side-effects [110]. D1, D2, and D3 receptors primarily control locomotor activity. Moreover, D1 and D2 receptors (and to a lesser extent D3) are essential in memory and learning mechanisms, mainly in the prefrontal cortex [111]. D2 receptors have a crucial function in psychotic behaviours since almost all effective antipsychotic drugs antagonize D2 receptors. The D3 receptor is primarily expressed in the limbic area of the brain [112]. D4 receptors are associated with relapse to stimulant use and selective D4 inhibitors/antagonists might be potential therapies for drug-relapse.

Outside the CNS, dopamine is also implicated in cardiovascular and renal functions, mainly through D1 and D2 receptors. Heterodimerization of dopamine receptors in various biological systems further complicates the role of dopaminergic interactions in PD [113]; therefore, designing more specific effective drugs using molecular docking might be a viable strategy to achieve drugs with fewer adverse effects in PD patients.

##### Gene Variants

Variants in a variety of genes have been reported to be associated with PD, including *SNCA* (encoding α-synuclein), *ADH1C, DJ-1, EIF4G1, FBXO7, GBA/GBAP1, GIGYF2, HTRA2, LRRK2* [114], *MAPT, PARK2, PARK7* [115], *PRKN, PINK1, PLA2G6, UCHL1*, and *VPS35* [116]. For example, several mutations to *LRRK2*, encoding the leucine-rich repeat kinase 2 (LRRK2), are associated with PD and it has been reported as a significant factor for drug resistance [117,118]. A panel of 160 kinase inhibitors was examined for their activity against LRRK2 in vitro employing a peptide substrate kinase assay and neuronal SH-SY5Y cells overexpressing LRRK2 [59]. In silico docking studies utilizing the LRRK2 kinase structure and some selected compounds found a correlation between docking scores for the LRRK2 ATP binding site and both in vitro and cellular compound activity [59].

##### Glutamate Antagonists

Glutamate receptors can be classified into two major classes: ionotropic (iGluRs) and metabotropic receptors (mGluRs). Glutamate antagonists have well-established neuroprotective effects through slowing the rate of dopaminergic neuron loss in the substantia nigra [119]. A number of glutamate antagonists improve motor function in PD animal models through acting on α-amino-3-hydroxy-5-methyl-4-isoxazole propionic acid (AMPA) and NMDA subtypes of ionotropic glutamate receptors. Nonetheless, systemic administration is associated with serious side-effects such as sedation and ataxia, especially for NMDA antagonists [120]. This has substantially affected their widespread use; therefore, developing selective antagonists against specific receptor isoforms that are preferentially expressed in the critical parts of the pathophysiological circuitry might be an interesting therapeutic approach in the future.

##### MAO-B

Monoamine oxidase inhibitors (MAOI) were one of the earliest drugs to be tried in PD and can be used with or without levodopa. Non-selective MAOI (such as tranylcypromine) have limited use in treating PD-associated depression due to their numerous side effects, while reversible and selective MAO-A inhibitors are more recommended. Selective and irreversible MAO-B inhibitors such as selegiline and rasagiline are recommended for the control of motor fluctuations and akinesia.

Selegiline is a selective, irreversible MAO-B inhibitor that has been widely used for PD treatment. It has been shown to delay the need for levodopa during early stages of PD and managing the end-of-dose akinesia in fully developed PD patients. A number of further irreversible and reversible MAO-B inhibitors have been developed.

Safinamide is a relatively new selective reversible MAO-B inhibitor with ion channel activity that does not cause a cheese-reaction, unlike other MAO-B inhibitors [121]. This drug enhances motor function in early PD [122].

### 3.3. Amyotrophic Lateral Sclerosis (ALS)

ALS is a lethal condition that is characterised by progressive muscular paralysis and wasting, reflecting degeneration of neurons controlling voluntary muscles, including both the upper motor neurons in the motor cortex and lower motor neurons in the brainstem and spinal cord [123].

Around 5000 people in the USA are diagnosed with ALS each year. Cumulatively, there are more than 30,000 and 5000 people affected with ALS in the USA and UK, respectively [124,125,126].

The pathogenesis of ALS is relatively poorly understood. Only two drugs are approved for ALS: one of these is a glutamate antagonist (riluzole) and the other (edaravone) works by an unknown mechanism.

#### 3.3.1. Macromolecular Targets in ALS

##### SOD1

Superoxide dismutase (SOD1) is an antioxidant enzyme involved in the detoxification of superoxide radicals. The SOD1 enzyme requires bound zinc and copper ions to maintain intra-molecular disulphide bonds [127]. Variation in zinc and copper ion binding to SOD1 leads to misfolded enzymes and can initiate aggregation and facilitate the protein instability associated with ALS.

In one study, 32,791 molecules were virtually screened by establishing an in silico assay system to screen for inhibitors of the aberrant interaction between mutant SOD1 and tubulin, with the aim of identifying lead compounds for ALS [128].

Molecular docking studies have been used to develop inhibitors of dimer destabilization and aggregation of the human SOD1 G85R mutant. CADD studies have predicted a number of inhibitors such as linear tripeptides [129], the tubulin binding site of G85R SOD1 [128], resveratrol [130], natural polyphenols of curcumin [131], kaempferol, and kaempferide [132] as potential lead compounds for treating ALS.

##### MAPK

Many processes within the cell, such as mitogenesis, apoptosis, oncogenesis, and differentiation, are associated with the mitogen activated protein kinases (MAPKs) [133]. MAPKs are activated by upstream kinases called MAPK kinases (termed MAPKK, MEK, or MKK) and an MAPK kinase kinase (termed MAPKKK, MEKK, or MKKK) [134], and are linked to the inhibition of proinflammatory cytokines [135]. An in silico and in vitro study of a MAPKK inhibitor (silibinin) used molecular docking to address the interactions of silibinin with p38 MAPK, which is an important kinase associated with glial cell activation and neuroinflammation [136].

##### Casein Kinase 1 (CK-1) Inhibitors

The protein kinase CK-1 was reported to directly phosphorylate Tyrosyl-DNA phosphodiesterase (TDP3). The latter is a DNA repair enzyme and is considered a promising target for antitumor and neurodegenerative therapy [137], and up-regulation of CK-1 is correlated with ALS [138]. Accordingly, CK-1δ inhibitors crossing the blood–brain barrier (BBB), such as riluzole and others, may be a novel approach to treat ALS [139,140,141].

##### Nav1.6 Sodium Channel

One of the most abundant sodium channels in the human brain is the voltage-gated sodium channel Nav1.6 [142,143]. Nav1.6 is a potential drug target for ALS as the blockage of these channels may enhance the survival of motor neurons in excitotoxic conditions [144,145,146]. In silico analyses demonstrated the interaction of riluzole with the Nav1.6 channel. Riluzole, an antiglutamatergic drug [147], exerts its antiglutamatergic effect partly by inactivation of Nav1.6 [63]. This suggests that riluzole reduces excitotoxicity via indirect interference with glutamate-mediated transmission [63]. The latter was proposed to participate in the loss of motor neurons resulting from a reduced glutamate uptake capacity of astrocytes in ALS [63,148].

### 3.4. Huntington’s Disease

HD is a genetic, incurable, and fatal neurodegenerative condition characterized by progressive degeneration of neurons, starting specifically with medium spiny neurons (MSNs) in the striatum, and leading to inevitable deterioration of the mental and physical abilities of those affected [149,150].

In the UK, the number of people diagnosed with HD is around 6000 people, whereas the number is around 30,000 in the USA [151,152,153,154].

HD is a monogenic disease caused by expansion of a CAG trinucleotide repeat in the *HTT* gene, leading to expansion of a polyglutamine tract in the Huntingtin protein, which is expressed ubiquitously throughout the brain. Mutant Huntingtin is prone to aggregation, but how this causes selective degeneration of striatal MSNs is poorly understood. Currently, no disease-modifying therapies or cures are available.

Reducing levels of mutant *HTT* is, understandably, a major therapeutic goal in HD. A recent study showed that intrathecal administration of the antisense oligonucleotide (ASO) IONIS-HTTRx (Tominersen) to HD patients resulted in a dose-dependent reduction of mutant HTT in the cerebrospinal fluid (CSF) [155]. Tominersen was rapidly moved to a Phase III trial. However, a press release by Roche in March 2021 announced the decision to discontinue dosing of Tominersen in manifest HD in the Phase III trial.

Despite the unfortunate news, these studies suggest that ASOs administration is a viable therapeutic strategy to reduce levels of toxic proteins in NDs. How and to what extent ASOs reach different parts of the central nervous system is not fully understood yet.

#### 3.4.1. Macromolecular Targets in HD

##### 4-Aminobutyrate Aminotransferase

4-Aminobutyrate aminotransferase (ABAT) (PDB ID: 1OHY) is responsible for the degradation of gamma-aminobutyric acid (GABA), a major inhibitory mediator for synaptic transmission in the mammalian CNS [156]. Reduction in GABAergic transmission is the result of many genetic disorders and chronic neurological diseases, including HD, AD, PD, and epilepsy. Unfortunately, GABA is unable to cross the BBB, preventing the direct use of exogenous GABA [157]. Enhancing the levels of GABA by decreasing its degradation by ABAT is an alternative strategy. In one study, the structures of thirty-two molecules from thirty-one medicinal plants were obtained from a chemical database and were chosen with the aid of previous literature reports. These 32 natural molecules were examined in a molecular docking study in which the researchers concluded that the top-ranked compounds may be suitable candidates for in vitro and in vivo studies of ABAT inhibition [158]. Moreover, GABA derivatives have been tested for ABAT binding in silico [156].

## 4. A Roadmap for Implementing CADD in ND Drug Design

Even with the number of successful implementations of CADD in modern drug discovery, it has its limitations. Molecules designed in silico utilizing computational and theoretical chemistry sometimes do not work in real biological systems [159,160]. In general, poor pharmacokinetics and/or pharmacodynamics result in only 40% of drug candidates passing phase I clinical trials [161]. Moreover, each computational technique depends on pre-determined algorithms that have their own limitations. CADD results must be validated in real biological systems, as many molecules that appear to bind in silico do not show the desired activity in vitro. Another limitation of CADD is that all tools for designing and discovery of new drugs are based on algorithms that, by necessity, simplify the underlying physics and chemistry and, therefore, have a variety of limitations that necessitate the continuous updating of these algorithms to enhance the accuracy and thus the provision of new drugs [162,163,164,165,166,167,168]. Furthermore, the shortage of experimental data regarding predicted absorption, distribution, metabolism, excretion, and toxicity results has led to several published failures [169,170,171,172,173].

To overcome the limitations and improve the accuracy of CADD it is necessary to update and develop software and associated algorithms, validate with experimental data, use reliable databases (e.g., PDB), and use algorithms that give docking scores that accurately predict in vitro binding with comprehensive and fully retrospective coverage of the published literature [174,175,176]. For example, by September 2020, the Cambridge Structural Dataset (CSD) acquired more than 1.8 million entries, which may help with future developments in small molecule structural modelling [177]. Consequently, the above-mentioned tools could help with future design of pharmacophores that possess the desired biological activity [178,179,180].

One of the main reasons for implementing in silico drug design is to predict the ligand–target binding in terms of binding site and binding strength. To predict potential ligands to treat NDs, novel target proteins must be identified and studied, and the resulting docking studies should be validated in vitro and eventually in the clinic [181,182,183].

In the meantime, there is no effective treatment to cure NDs, although many treatments are available that offer minor improvement of symptoms [2]. The development of effective treatments is further hindered by the BBB that excludes many molecules from the CNS parenchyma [184,185,186]. Accordingly, clinical effectiveness of a potential drug is not guaranteed even with positive data in silico, in vitro, and in vivo [187,188,189,190].

New experimental approaches including genome-wide association studies (GWAS) [188,191,192], CRISPR-Cas9 technology [193,194,195], high throughput screening (HTS) [196], organ-on-chip technologies [197,198], functional MRI (fMRI) techniques [199,200], and positron emission tomography (PET) [201] may lead to new drug targets for NDs, which can feed into future CADD projects.

Being incurable, the NDs are major challenges to healthcare providers and research scientists. The accelerating increase in the numbers of affected people adds more impetus to tackle NDs. Developing a better understanding of NDs and the underlying molecular pathophysiology will provide more opportunities to develop novel treatments in the near future. This may be achieved with the incorporation of computational tools. CADD can have a major impact on drug discovery by saving both time and money and reducing the risk of following up with the development of non-viable leads.

## Figures and Tables

**Table 1:** The 30 most highly-scored pieces of software for CADD. Software was ranked according to the equation S*_i_* = log(10^6^.C*_i_*/C_total_) where S*_i_* is the score for tool *i*, C*_i_* is the number of citations to tool *i*, and C_total_ is the number of citations to all tools considered. Number of citations was obtained using Google Scholar, last accessed on 14 April 2021.

No.	Software	No. of Citations to Published Studies	Score	Features	Accessibility	Website
1	HADDOCK	26,490	4.7323	Docks protein−protein based on biochemical or biophysical information	Free	https://wenmr.science.uu.nl/haddock2.4/
2	AutoDock Autodock 1 Autodock 2.4 Autodock 3 Autodock 4 Autodock 4.2 Autodock Vina AutoDockFR AutoDockTools	22,422	4.6599	Automated docking tools	Free	http://autodock.scripps.edu/
3	Glide Glide 1.8 Glide 2 Glide 2.5	22,091	4.6535	Rapid, accurate docking and scoring approach	Subscription	https://www.schrodinger.com/glide
4	FlexX	19,987	4.6100	Predicts the geometry of the protein–ligand complex and estimates the binding affinity	Free	https://www.biosolveit.de/FlexX/
5	LigandFit	19,890	4.6079	Presents a shape-based approach for docking ligands into the active site of the protein	Subscription	https://www.phenix-online.org/documentation/reference/ligandfit.html
6	AmberTools	14,572	4.4728	A suite of biomolecular simulation programs	Subscription	https://ambermd.org/
7	ENCoM	13,145	4.4280	A coarse-grained normal mode analysis method utilized for different residues in proteins or nucleotides in RNA	Free	http://biophys.umontreal.ca/nrg/resources.html
8	PROCHECK-NMR	10,783	4.3420	Checks the stereochemical quality of a protein structure solved by NMR	Free	https://www.ebi.ac.uk/thornton-srv/software/PROCHECK/
9	MCDOCK	10,603	4.3347	Allows for a full flexibility of ligands in the docking calculations	Free	DOI: 10.1021/jm990129n
10	ICM ICM 2.8 ICM-Dock	10,271	4.3209	A new method for protein modelling and design applications to docking and structure prediction	Subscription	http://www.molsoft.com/docking.html
11	Dock Dock2 Dock3 Dock4 Dock5 Dock6 Dock7 Dock8 Dock9	8181	4.2221	Based on a geometric matching algorithm	Free	http://dock.compbio.ucsf.edu/
12	SOFT Docking	7474	4.1828	Predicts the sites of interaction between two cognate molecules based on their 3D structures	Subscription	https://doi.org/10.1016/0022-2836(91)90859-5
13	FDS	7188	4.1659	Cluster analysis based on distance similarities	Free	http://www.scfbio-iitd.res.in/dock/fds.jsp
14	DockVision	6950	4.1512	Increases capability to generate laudable results	Free	http://dockvision.sness.net/overview/overview.html
15	PRODOCK	6442	4.1183	Renders the programming easier and the definition of molecular flexibility more straightforward	Subscription	https://doi.org/10.1002/(SICI)1096-987X(199903)20:4<412::AID-JCC3>3.0.CO;2-N
16	YASARA YASARA Dynamics YASARA Model YASARA NMR Module YASARA Structure YASARA View YASARA Virtual Reality Workstation YASARA/WHAT IF Twinset	5870	4.0779	A molecular-graphics, -modelling, and -simulation program	Free	http://www.yasara.org/products.htm
17	KBDOCK	5820	4.0742	A program that proposes structural templates for protein docking	Free	http://kbdock.loria.fr/
18	TreeDock	5796	4.0724	A docking tool that is able to explore all clash-free orientations at very fine resolution in a reasonable time	Subscription	https://doi.org/10.1021/ja011240x
19	LePro	5639	4.0605	Generates a docking input file for LeDock with refined protein atoms within 0.4 nm of any atom of the ligand	Free	http://www.lephar.com/download.htm
20	DockoMatic	5594	4.0570	A software that docks secondary ligands, used to assist inverse virtual screening	Free	https://doi.org/10.1186/1756-0500-3-289
21	SYBYL_ChemScore SYBYL_D-Score SYBYL_F-Score SYBYL_G-Score	5486	4.0485	A conformational sampling and scoring function	Subscription	https://doi.org/10.1021/jm0203783
22	ZDOCK ZDOCKpro	5415	4.0429	A new scoring function for the initial stage of unbound docking	Subscription	http://zdock.umassmed.edu/
23	AADS	5087	4.0157	An automated active site identification, docking, and scoring protocol	Free	http://www.scfbio-iitd.res.in/dock/ActiveSite_new.jsp
24	Surflex Dock	4896	3.9991	An automatic and flexible molecular docking algorithm for rapid in silico drug-screening applications	Subscription	https://doi.org/10.1007/s10822-007-9114-2
25	PyMOL PyMOL 1.4.1 PyMOL 2.1.1 PyMOL 2.4	4805	3.9910	An open-source, user-sponsored, molecular visualization system	Subscription	http://www.pymol.org
26	FlipDock	4614	3.9733	Allows the automated docking of flexible ligand molecules into active sites of flexible receptor molecules	Free	http://flipdock.scripps.edu/
27	SymmDock	4545	3.9668	A flexible induced-fit backbone refinement in molecular docking	Free	http://bioinfo3d.cs.tau.ac.il/FiberDock/php.php
28	ClusPro	4360	3.9487	A widely used tool for protein–protein docking	Free	http://nrc.bu.edu/cluster
29	Surflex	4180	3.9304	A robust screening tool	Subscription	https://pubmed.ncbi.nlm.nih.gov/12570372/
30	ConsDock	4001	3.9114	A pose within 2 A^o^ RMSD of the X-ray structure can be performed with this software	Subscription	https://doi.org/10.1002/prot.10119

**Table 2:** NDs with specified molecular targets and selected examples of drugs that have been identified with the aid of in silico drug design. The assay format used to validate each drug is indicated and drugs that progressed to clinical trials are highlighted in bold.

NDs	Molecular Docking Targets	Molecule	Software	Assay Type
Alzheimer’s disease	Acetylcholinesterase, Beta-secretase enzymes, Muscarinic and nicotinic ACh receptors, N-methyl-D-aspartate receptor, Tau proteins	1-benzy-l1,2,3,4-tetrahydro- b-carboline), 3-substituted-1H-indoles, 6-triazolyl amidine derivatives [40]	ICM	cell-based assay [40]
		Chloropyridonepezil [41]	Autodock Vina	In vitro blood–brain barrier model [42]
		Flavone, 5-hydroxyflavone, 7-hydroxyflavone, chrysin, apigenin, kaempferol, fisetin, and quercetin [43]	AutoDock	Mice and rats models [44,45]
		Ifenprodil [46]	Schrödinger Suite	Primary cultures from chicken embryo forebrain (E10) [46]
		Memantine [47,48]	Glide	Human clinical trial [49]
		Morin [50]	Glide	In APPswe/PS1dE9 mice [51]
		Pyridopyrimidine derivatives [52]	Auto grid and auto dock	In vitro enzyme inhibitory model [53]
		Pyridonepezil [54]	Autodock Vina	In vitro blood–brain barrier model [42]
		Piperazine derivatives [55]	PASS software	Tested on AChE in vitro by using Ellman’s method [56]
		Rutin [57]	AutoDock and Autodock Vina	Doxorubicin (DOX)-treated neuroblastoma cells (IMR32) and doxorubic-induced cognitive dysfunction in Wistar rats [58]
Parkinson’s disease	Dopamine receptors, expression and mitochondrial localization, Mutant LRRK2, Mutated, PINK1, PARK2, DJ1 SNCA Motif	LRRK2 kinase inhibitors (9-methyl-N-phenylpurine-2,8-diamine, N-phenylquinazolin-4-amine, and 1,3-dihydroindol-2-one) [59]	MOE	Both in vitro and in vivo studies were established [60]
Amyotrophic lateral sclerosis	Mutant SODI, SODI oligomerization, CASP-3, CASP-8, TDP-43, p38 MAPK Nav1.6 sodium channel	Angiogenin [61]	AmberTools20	HeLa cells (Nuclear translocation assay) [61])
		Hesperidin and THSG [62])	(Molecular Dynamics (MD) Simulation	High affinity to mutant SOD1 [62]
		Riluzole [63]	PROCHECK program	FDA-approved drug for ALS [64]
Huntington’s disease	FIP-2 Specificity protein, 1HTT Interacting proteins Mutant HTT, Infant Testing Nuclear receptor corepressor, Postsynaptic density-95	T1–11 (synthesized in a high yield by the substitution reaction) [65]	AutoDockTools	PC12 cells [65]

## Data Availability

Not applicable.

## Supplementary Materials

The following are available online at https://www.mdpi.com/article/10.3390/ijms22094688/s1.
