# Supplementary material for: Advances in Applying Computer-Aided Drug Design for Neurodegenerative Diseases
Source: Int J Mol Sci. 2021 Apr 28;22(9):4688. doi: 10.3390/ijms22094688 (PMC8124449; doi:10.3390/ijms22094688)
Supplement: Supplementary file 1 [file ijms-22-04688-s001.zip › ijms-1181389-supplementary.pdf]

Supplementary Table S1: A list of 211 pieces of software for CADD. Software was ranked according to the equation $S_i = \log(10^6 . C_i / C_{total})$ where S_i is the score for tool *i*, C_i is the number of citations to tool *i*, and C_{total} is the number of citations to all tools considered. Number of citations was obtained using Google Scholar, last accessed on 14th April 2021.

No.	Software	No. of citations to published studies	Score	Features	Accessibility	Website
1	HADDOCK	26490	4.7323	Docks protein–protein based on biochemical or biophysical information	Free	https://wenmr.science.uu.nl/haddock2.4/
2	AutoDock Autodock 1 Autodock 2.4 Autodock 3 Autodock 4 Autodock 4.2 Autodock Vina AutoDockFR AutoDockTools	22422	4.6599	Automated docking tools	Free	http://autodock.scripps.edu/
3	Glide Glide 1.8 Glide 2 Glide 2.5	22091	4.6535	Rapid, Accurate Docking and Scoring approach	Subscription	https://www.schrodinger.com/glide
4	FlexX	19987	4.6100	Predicts the geometry of the protein-ligand complex and estimates the binding affinity	Free	https://www.biosolveit.de/FlexX/
5	LigandFit	19890	4.6079	Presents a shape-based approach for docking ligands into the active site of the protein	Subscription	https://www.phenix-online.org/documentation/reference/ligandfit.html
6	AmberTools	14572	4.4728	A suite of biomolecular simulation programs	Subscription	https://ambermd.org/
7	ENCoM	13145	4.4280	A coarse-grained normal mode analysis method utilized for different residues in proteins or nucleotides in RNA	Free	http://biophys.umontreal.ca/nrg/resources.html
8	PROCHECK-NMR	10783	4.3420	Checks the stereochemical quality of a protein structure solved by NMR	Free	https://www.ebi.ac.uk/thornton-srv/software/PROCHECK/

No.	Software	No. of citations to published studies	Score	Features	Accessibility	Website
9	MCDOCK	10603	4.3347	Allows for a full flexibility of ligands in the docking calculations	Free	DOI: 10.1021/jm990129n
10	ICM ICM 2.8 ICM-Dock	10271	4.3209	A new method for protein modelling and design applications to docking and structure prediction	Subscription	http://www.molsoft.com/docking.html
11	Dock Dock2 Dock3 Dock4 Dock5 Dock6 Dock7 Dock8 Dock9	8181	4.2221	Based on Geometric Matching Algorithm	Free	http://dock.compbio.ucsf.edu/
12	SOFT Docking	7474	4.1828	Predicts the sites of interaction between two cognate molecules based on their 3D structures	Subscription	https://doi.org/10.1016/0022-2836(91)90859-5
13	FDS	7188	4.1659	Cluster analysis based on distance similarities	Free	http://www.scfbio-iitd.res.in/dock/fds.jsp
14	DockVision	6950	4.1512	Increases capability to generate laudable results	Free	http://dockvision.sness.net/overview/overview.html
15	PRODOCK	6442	4.1183	Renders the programming easier and the definition of molecular flexibility straighter forward	Subscription	https://doi.org/10.1002/(SICI)1096-987X(199903)20:4<412::AID-JCC3>3.0.CO;2-N
16	YASARA YASARA Dynamics YASARA Model YASARA NMR Module YASARA Structure YASARA View YASARA Virtual Reality Workstation YASARA/WHAT IF Twinset	5870	4.0779	A molecular-graphics, -modeling and -simulation program	Free	http://www.yasara.org/products.htm
17	KBDOCK	5820	4.0742	A program that proposes structural templates for protein docking	Free	http://kbdock.loria.fr/

No.	Software	No. of citations to published studies	Score	Features	Accessibility	Website
18	TreeDock	5796	4.0724	A docking tool that is able explore all clash-free orientations at very fine resolution in a reasonable time	Subscription	https://doi.org/10.1021/ja011240x
19	LePro	5639	4.0605	Generates docking input file for LeDock with refined protein atoms within 0.4 nm of any atom of the ligand	Free	http://www.lephar.com/download.htm
20	DockoMatic	5594	4.0570	A software that docks secondary ligands used to assist inverse virtual screening	Free	https://doi.org/10.1186/1756-0500-3-289
21	SYBYL_ChemScore SYBYL_D-Score SYBYL_F-Score SYBYL_G-Score	5486	4.0485	A conformational sampling and scoring function	Subscription	https://doi.org/10.1021/jm0203783
22	ZDOCK ZDOCKpro	5415	4.0429	A new scoring function for the initial stage of unbound docking	Subscription	http://zdock.umassmed.edu/
23	AADS	5087	4.0157	An automated active site identification, docking, and scoring protocol	Free	http://www.scfbio-iitd.res.in/dock/ActiveSite_new.jsp
24	Surflex Dock	4896	3.9991	An automatic and flexible molecular docking algorithm for rapid in-silico drug-screening applications	Subscription	https://doi.org/10.1007/s10822-007-9114-2
25	PyMOL PyMOL 1.4.1 PyMOL 2.1.1 PyMOL 2.4	4805	3.9910	An open-source, user-sponsored, molecular visualization system	Subscription	http://www.pymol.org
26	FlipDock	4614	3.9733	Allows the automated docking of flexible ligand molecules into active sites of flexible receptor molecules	Free	http://flipdock.scripps.edu/
27	SymmDock	4545	3.9668	A Flexible Induced-fit Backbone Refinement in Molecular Docking	Free	http://bioinfo3d.cs.tau.ac.il/FiberDock/php.php
28	ClusPro	4360	3.9487	A widely used tool for protein-protein docking	Free	http://nrc.bu.edu/cluster

No.	Software	No. of citations to published studies	Score	Features	Accessibility	Website
29	Surflex	4180	3.9304	A robust screening tool	Subscription	https://pubmed.ncbi.nlm.nih.gov/12570372/
30	ConsDock	4001	3.9114	A pose within 2 A° RMSD of the X-ray structure can be performed with this software	Subscription	https://doi.org/10.1002/prot.10119
31	EADock	3961	3.9071	A software that is based on "evolutionary algorithms" that generates the good solution through sampling and recognizes this solution as the correct one by its scoring function	Free	http://www.swissdock.ch/
32	Fred	3953	3.9062	A program that docks 70 % of the structures within 2 Å in the cognate docking test	Subscription	https://www.eyesopen.com/oedocking-tk
33	PASS	3907	3.9011	A prediction of Activity Spectra for Substances	Free	http://www.way2drug.com/passonline/
34	PhDock	3890	3.8992	A determination of target-based theoretical pharmacophore	Free	http://iscreen.cmu.edu.tw/phdock.php
35	Robetta	3545	3.8589	A protein structure prediction service that is continually evaluated through Continuous Automated Model evaluatiOn (CAMEO)	Subscription	http://robetta.bakerlab.org/
36	InterEvDock	3461	3.8485	An expanded server for protein docking using evolutionary and biological information from homology models and multimeric inputs	Free	https://mobyle.rpbs.univ-paris-diderot.fr/cgi-bin/portal.py#forms::InterEvDock2
37	FlexDock	3433	3.8449	An algorithm for Automatic prediction of protein interactions with large scale motion	Subscription	http://bioinfo3d.cs.tau.ac.il/
38	RiboDock	3316	3.8299	A virtual-screening system for automated flexible docking.	Free	https://doi.org/10.1023/B:JCAM.0000035199.48747.1e
39	GASP	3271	3.8239	A "Genetic Algorithm Similarity Program" used for testing and investigating methods in statistical genetics by generating samples of	Subscription	https://doi.org/10.1023/A:1008844323931

No.	Software	No. of citations to published studies	Score	Features	Accessibility	Website
				family data based on user specified models		
40	GOLD	3208	3.8155	A protein ligand docking software that enables you to make confident binding mode predictions, and achieve high database enrichments	Subscription	https://doi.org/10.1002/prot.10465
41	FTDOCK	3205	3.8151	A tool that performs a global scan of translational and rotational space	Free	http://www.sbg.bio.ic.ac.uk/docking/download.html
42	QXP	3186	3.8125	A quick explore (QXP) search algorithm	Subscription	https://doi.org/10.1023/A:1007907728892
43	GlamDock	3150	3.8076	A mapping-based description of the rigid body translation and rotation	Subscription	http://www.chil2.de/Glamdock.html
44	PSO@AUTODOCK	3113	3.8024	A Fast and efficient protein ligand docking program based on swarm optimization	Subscription	https://doi.org/10.1111/j.1747-0285.2007.00588.x
45	SKE-DOCK	3077	3.7974	A simple geometry docking and a knowledge base scoring function	Subscription	http://www.pharm.kitasato-u.ac.jp/bmd/files/SKE_DOCK.html
46	Q-Dock(LHM)	3069	3.7963	A reduced representation mod description	Subscription	DOI: 10.1002/jcc.21395
47	PatchDock	2931	3.7763	An object recognition and image segmentation techniques used in computer vision	Subscription	http://bioinfo3d.cs.tau.ac.il/wk/index.php/Servers_%26_Software
48	MS-DOCK	2804	3.7570	All multiple conformations are rigidly docked	Subscription	https://doi.org/10.1186/1471-2105-9-184
49	PSI-DOCK	2638	3.7305	A docking method, Pose-Sensitive Inclined (PSI)-DOCK, for flexible ligand docking	Subscription	https://doi.org/10.1002/prot.20790

No.	Software	No. of citations to published studies	Score	Features	Accessibility	Website
50	PostDOCK	2559	3.7173	A tool that distinguishes true binding ligand-protein complexes from docking artifacts	Subscription	https://doi.org/10.1021/jm0493360
51	MolDock	2553	3.7163	Combining a differential evolution within a cavity prediction algorithm	Subscription	https://doi.org/10.1021/jm051197e
52	MEDock	2510	3.7089	A worldwide search strategy that exploits the highest entropy attribute of the Gaussian probability distribution	Free	https://medock.ee.ncku.edu.tw/
53	SwarmDock	2449	3.6982	A flexible protein-protein docking method	Free	https://bmm.crick.ac.uk/~svc-bmm-swarmdock/submit.cgi
54	GAsDock	2373	3.6846	A rapid, accurate, and flexible docking program	Subscription	doi:10.1016/j.bmcl.2004.06.091
55	SDOCKER	2331	3.6768	A method utilizing available x-ray structures to improve docking accuracy	Free	https://github.com/xogeny/sdocker
56	SOFTDOCK	2279	3.6670	A model continuity across development lifecycle	Subscription	DOI: 10.1109/DEXA.1999.795265
57	DOCK Blaster	2265	3.6643	A free web-based service for molecular docking and virtual screening	Subscription	http://blaster.docking.org
58	M-ZDOCK	2257	3.6628	A symmetric multimer docking	Subscription	http://zdock.umassmed.edu/m-zdock/
59	GAPDOCK	2249	3.6612	A molecular docking methodology explores the behaviour of small molecules in the binding site of a target protein	Subscription	https://doi.org/10.1002/prot.10386
60	3D-QSAR	2232	3.6580	Simple and advanced tools for developing QSAR models	Free	https://www.3d-qsar.com/
61	INVDOCK	2221	3.6558	A computer-automated identification of potential protein and nucleic acid (RNA or DNA) targets of a small molecule	Subscription	http://bidd.nus.edu.sg/group/softwares/invdock.htm

No.	Software	No. of citations to published studies	Score	Features	Accessibility	Website
62	MOE	2216	3.6548	An integrated computer-aided molecular design platform	Subscription	https://www.chemcomp.com/Products.htm
63	rDock rDock 3.0	2191	3.6499	A docking tool for small molecules against proteins and nucleic acids	Free	http://rdock.sourceforge.net/
64	DOCKovalent	2010	3.6125	Screens large virtual libraries of electrophilic small molecules	Free	http://covalent.docking.org/
65	TarFisDock	1930	3.5948	A web server that docks small molecules with protein structures in the Potential Drug Target Database (PDTD) in an effort to discover new drug targets	Subscription	https://bio.tools/tarfisdock#!
66	SDOCK	1927	3.5941	Improves the efficiency and robustness of particle swarm optimization (PSO) for solving flexible protein-ligand docking problems	Subscription	https://onlinelibrary.wiley.com/doi/abs/10.1002/jcc.21839 http://mdl.ipc.pku.edu.cn/cgi-bin/down.cgi
67	FINDSITE-LHM	1926	3.5939	A homology modelling approach to flexible ligand docking that uses a collection of common molecule substructures derived from evolutionarily related templates as the reference compounds in similarity-based ligand binding pose prediction	Free	http://www.mybiosoftware.com/findsite-lhm-1-0-homology-modeling-approach-flexible-ligand-docking.html
68	DAIM-SEED-FFLD	1894	3.5866	DAIM (Decomposition and Identification of Molecules), SEED (Solvation Energy for Exhaustive Docking), and FFLD (Fragment-based Flexible Ligand Docking)	Free	http://www.biochem-caflisch.uzh.ch/movies/0003
69	FlexPepDock	1891	3.5859	Able to refine a coarse starting structure of a peptide-protein complex to a near-native model of the interaction	Free	http://flexpepdock.furmanlab.cs.huji.ac.il/
70	VoteDock	1860	3.5788	Provides fast and accurate prediction method for 3D structure of a protein–ligand complex	Subscription	https://connects.catalyst.harvard.edu/Profiles/display/1104658
71	CDocker	1846	3.5755	Molecular dynamics (MD) simulated-annealing-based algorithm.	Free	https://doi.org/10.1002/jcc.10306

No.	Software	No. of citations to published studies	Score	Features	Accessibility	Website
72	POSIT	1792	3.5626	A new approach to structure-based drug design (POSIT) rigorously built on the simple concept that pose prediction is intimately coupled to the quality and availability of experimental structural data	Free	https://docs.eyesopen.com/applications/oedocking/posit/posit_overview.html
73	DynaDock	1709	3.5420	Used for docking peptides into flexible receptors	Free	https://doi.org/10.1002/prot.22629
74	PepCrawler	1700	3.5397	A fast RRT-based algorithm for high-resolution refinement and binding-affinity estimation of peptide inhibitors	Free	http://bioinfo3d.cs.tau.ac.il/PepCrawler/php.php
75	GEMDOCK<br>GemDock 1.0<br>GemDock 2.0<br>GemDock 2.1<br>GemDock cent OS5<br>GemDock linux 9<br>GemDock-windos	1666	3.5309	Uses a Generic Evolutionary Method for molecular docking (GEMDOCK) and an empirical scoring function	Free	http://gemdock.life.nctu.edu.tw/dock/
76	FireDock	1654	3.5278	An efficient method for refinement and re-scoring of rigid-body protein-protein docking solutions	Free	http://bioinfo3d.cs.tau.ac.il/FireDock/
77	CovalentDock	1648	3.5262	Used for covalent bonds	Subscription	https://doi.org/10.1002/jcc.23136
78	pyDock	1644	3.5252	A program which was implemented in order to check the scoring of rigid-body docking poses	Subscription	https://life.bsc.es/pid/pydock/get_pydock.html
79	DOCKGROUND	1629	3.5212	Implements a comprehensive database of co-crystallized (bound–bound) protein–protein complexes	Free	http://dockground.bioinformatics.ku.edu/
80	SODOCK	1564	3.5035	Based on particle swarm optimization (PSO) for solving flexible protein–ligand docking problems	Free	http://www.mybiosoftware.com/tag/sodock
81	SwissDock	1476	3.4783	A web-server program dedicated to the docking of small molecules on target proteins	Free	http://www.swissdock.ch/docking

No.	Software	No. of citations to published studies	Score	Features	Accessibility	Website
82	MedusaDock	1471	3.4769	Models ligand and receptor flexibility simultaneously with sets of discrete rotamers	Free	https://dokhlab.med.psu.edu/medusadock/#/NewTask
83	FiberDock	1427	3.4637	An efficient method for flexible refinement and re-scoring of rigid-body protein-protein docking solutions	Free	http://bioinfo3d.cs.tau.ac.il/FiberDock
84	VSDMIP	1426	3.4634	Virtual screening data management on an integrated platform	Free	https://ub.cbm.uam.es/software/vsdmip/
85	GalaxySite	1426	3.4634	An installation of the Galaxy software combined with many common tools and data	Free	http://galaxy.seoklab.org/site
86	FlexX-Scan	1365	3.4444	Developed to enhance the speed of the virtual screening process	Subscription	https://pubmed.ncbi.nlm.nih.gov/15382244/
87	Kinome Render	1346	3.4383	Produces customized annotations on the human kinome tree	Free	http://biophys.umontreal.ca/nrg/resources.html
88	Fleksy	1333	3.4341	An approach to consider both ligand and receptor flexibility in small molecule docking	Subscription	https://doi.org/10.1021/jm070593p
89	KinDock	1319	3.4295	A web server for the analysis of ATP-binding sites of protein kinases	Obsolete	http://abcis.cbs.cnrs.fr/kindock/
90	PharmDock	1306	3.4252	A pharmacophore-based docking program that combines pose sampling and ranking based on optimized protein-based pharmacophore models	Free	https://www.ncbi.nlm.nih.gov/pmc/articles/PMC4012150/
91	GriDock	1284	3.4178	Designed to perform the molecular dockings of a large number of ligands stored in a single database (in a format supported by VEGA)	Free	https://www.ddl.unimi.it/cms/index.php?Software_projects:GriDock
92	ParaDockS	1282	3.4171	Designed to hold different optimization algorithms and objective functions and provides an accurate prediction of protein–DNA complexes	Subscription	https://doi.org/10.1021/ci900467x

No.	Software	No. of citations to published studies	Score	Features	Accessibility	Website
93	EpiDOCK	1270	3.4131	The first structure-based server that predicts binding to the 23 most frequent MHC class II binding proteins	Free	http://www.ddg-pharmfac.net/epidock/
94	MacDOCK	1248	3.4055	Able to generate orientations consistent with the known covalent binding mode of these complexes	Subscription	DOI: 10.1007/s10822-004-5291-4
95	FIPSDock	1239	3.4023	Implements a variant of the Fully Informed Particle Swarm (FIPS) optimization method	Subscription	http://hub.hku.hk/handle/10722/189104
96	Flexidock	1239	3.4023	Brings the convenience of a HDD docking station, with the performance of a HDD mobile rack	Subscription	http://scholar.dkyobobook.co.kr/searchDetail.laf?barcode=4010024416172
97	Smina	1230	3.3992	A general methodology for designing an empirical scoring function	Free	https://pypi.org/project/docking-py/
98	BetaDock	1228	3.3985	A molecular docking simulation software based on the theory of Beta-complex	Free	DOI: 10.1080/07391102.2011.10507384
99	FLOG	1215	3.3938	Flexible Ligands Oriented on Grid that searches a database of 3D coordinates to explore molecules complementary to a macromolecular receptor of known 3D structure	Subscription	https://doi.org/10.1007/BF00119865
100	Combibuild	1206	3.3906	Builds combinatorial libraries for CRISPR screening	Subscription	https://doi.org/10.1021/jm010338j
101	MoDock	1174	3.3789	A main method of structure-based virtual screening	Subscription	https://doi.org/10.1186/s13015-015-0034-8
102	CRDOCK	1173	3.3786	Contains a search engine, several energy minimization algorithms, and different scorings	Free	DOI: 10.1021/ci300194a
103	FRODOCK	1154	3.3715	Fast rotational docking based on FRM (Fast Rotational Method) to perform protein-protein docking	Free	http://frodock.chaconlab.org/

No.	Software	No. of citations to published studies	Score	Features	Accessibility	Website
104	GalaxyDock GalaxyDock1 GalaxyDock2 GalaxyDock3 GalaxyDock BP2	1154	3.3715	Allows flexibility of pre-selected side-chains of ligand using Conformational Space Annealing	Free	http://galaxy.seoklab.org/softwares/galaxydock.html
105	SANDOCK	1153	3.3711	Uses point complementary method that is based on shape and chemical complementarity between interacting molecules	Subscription	DOI: 10.1006/jmbi.1997.1608
106	VSDocker	1143	3.3673	Allows using autodock4 for optimized virtual ligand screening on computer clusters	Subscription	https://www.hsls.pitt.edu/obrc/index.php?page=URL1275058861
107	EUDOC	1114	3.3561	A computer program for identification of drug interaction sites in macromolecules and drug leads from chemical databases	Subscription	https://doi.org/10.1002/jcc.1129
108	PyRX PyRX 0.5 PyRX 0.7 PyRX 0.8 PyRX 0.9.6 PyRX 0.9.8	1085	3.3447	A tool for Rational Drug Design	Free & Subscription	https://pyrx.sourceforge.io/
109	CombDock	1078	3.3419	A combinatorial docking algorithm for the structural units-assembly problem	Subscription	http://bioinfo3d.cs.tau.ac.il/wk/index.php/Servers_%26_Software
110	FastDock	1044	3.3280	Lamarckian genetic algorithm to estimate the binding affinities between the structures	Free	https://pypi.org/project/fastdock/
111	Lead finder Leadfinder 2.3 Leadfinder 3.8	1029	3.3217	Provides a fast rate of calculations with unique scoring function that provides unsurpassed accuracy of calculations	Subscription	https://www.cresset-group.com/software/lead-finder/
112	pyDockTET	1009	3.3131	A tethered-docking program which uses rigid-body docking system to generate domain-domain poses	Subscription	DOI: 10.1186/1471-2105-9-441
113	MADAMM	1008	3.3127	Utilizes rotamer libraries to produce several combinations of conformers	Subscription	https://doi.org/10.1002/prot.22146
114	pyDockRST	1003	3.3106	Uses the percentage of satisfied restrains	Subscription	DOI: 10.1016/j.jmb.2006.01.001

No.	Software	No. of citations to published studies	Score	Features	Accessibility	Website
115	DOCKTITE	995	3.3071	Utilized for covalent docking in the Molecular Operating Environment (MOE)	Subscription	https://pubs.acs.org/doi/10.1021/ci500681r
116	pyDockCG	992	3.3058	A coarse-grained potential for protein-protein docking scoring and refinement, based on the known UNRES model for polypeptide chains	Subscription	https://doi.org/10.1021/jp112292b
117	ASEDock	990	3.3049	Based on a shape similarity assessment between a concave portion and the ligand	Subscription	https://doi.org/10.1021/ci700352q
118	IsoMIF	975	3.2983	Allows to identify binding site molecular interaction field (MIF) similarities between pairs of structures	Free	http://biophys.umontreal.ca/nrg/resources.html
119	LigDockCSA	975	3.2983	Combines a highly efficient search method with a scoring function based on the autodock energy function with a piecewise linear potential (PLP) torsional energy	Subscription	https://doi.org/10.1002/jcc.21905
120	GalaxyPepDock	946	3.2851	A web server for protein structure prediction, refinement, and related methods Protein-peptide docking based on interaction similarity	Free	http://galaxy.seoklab.org/pepdock
121	pyDockWEB	924	3.2749	A web server for the rigid- body docking forecast of protein–protein complex structures using an updated version of the pydock scoring algorithm	Free	https://life.bsc.es/pid/pydockweb
122	AnchorDock	912	3.2693	Automatically identifying anchoring spots on the protein surface	Subscription	https://doi.org/10.1016/j.str.2015.03.010
123	ArgusLab	883	3.2552	A molecular modelling graphics and drug design program for Windows OS	Free	http://www.arguslab.com/arguslab.com/ArgusLab.html
124	idock	882	3.2547	A multithreaded virtual screening tool for flexible ligand docking for computational drug discovery	Subscription	https://GitHub.com/HongjianLi/idock
125	HybridDock	854	3.2407	Significantly improves the performance in both binding affinity and binding mode predictions, compared to the sole mdock program	Subscription	https://doi.org/10.1021/acs.jcim.5b00275

No.	Software	No. of citations to published studies	Score	Features	Accessibility	Website
126	ParaDock	840	3.2335	Rigid protein flexible DNA Docking	Free	http://bioinfo3d.cs.tau.ac.il/ParaDock/php.php
127	WinDock	810	3.2177	Designed to help researchers perform structure-based drug discovery tasks	Subscription	https://doi.org/10.1002/jcc.20756
128	OptiDock	787	3.2052	Involves choosing a representative subset of compounds	Subscription	https://doi.org/10.1021/cc034068x
129	BiGGER	765	3.1929	Predicts the mode of binding between two proteins	Free	https://doi.org/10.1002/(SICI)1097-0134(20000601)39:4<372::AID-PROT100>3.0.CO;2-Q
130	MTiOpenScreen	759	3.1895	Automated ligand docking into entire protein surface for up to 10 ligands allowing to detect druggable cavities using autodock 4.2	Free	http://bioserv.rpbs.univ-paris-diderot.fr/services/MTiOpenScreen/
131	HomDock	752	3.1855	Improves docking accuracy and efficiency	Subscription	http://www.chil2.de/HomDock.html
132	BDOCK	745	3.1814	An enzyme-inhibitor docking algorithm based on the degree of burial and conservation of surface residues	Free	www.biotec.tu-dresden.de /approximately bhuang/bdock
133	LiGendock	730	3.1726	Based on pharmacophore models of binding sites, including a non-enumerative docking calculation	Subscription	https://doi.org/10.1021/ci400079k
134	DockBench	726	3.1702	Handles seven docking software packages and offers the possibility to test up to seventeen different protocols	Free	doi: 10.3390/molecules20069977
135	AUDocker LE	673	3.1373	Designed to develop a software tool as a front-end graphical interface	Free	DOI: 10.1186/1756-0500-4-445
136	3dRPC	667	3.1334	Developed as an automated method for predicting 3D structures of RNA-protein	Free	http://biophy.hust.edu.cn/3dRPC.html

No.	Software	No. of citations to published studies	Score	Features	Accessibility	Website
137	DarwinDock	665	3.1321	Isolates posing and scoring into separate stages to allow complete binding site sampling	Subscription	https://resolver.caltech.edu/CaltechTHESIS:06122017-230026717
138	VLifeMDS	648	3.1208	An integrated platform for Computer Aided Drug Design (CADD) and molecular discovery	Subscription	https://www.vlifesciences.com/products/VLifeMDS/Product_VLifeMDS.php
139	pDOCK	630	3.1086	An experimental pygtk docking implementation	Free	https://pypi.org/project/pdock/
140	CovalentDock Cloud	621	3.1023	Provides a simple user-friendly web interface to perform covalent docking	Free	http://docking.sce.ntu.edu.sg/.
141	SLIM (BSP-SLIM) SLIM21	613	3.0967	A blind molecular docking method on low-resolution protein structures	Free	https://zhanglab.ccmb.med.umich.edu/BSP-SLIM/
142	3dRNAscore	590	3.0801	Developed as an automated method for predicting 3D structures of noncoding RNAs	Free	http://biophy.hust.edu.cn/3dRNAscore.html
143	FlexAID	588	3.0786	Can use small-molecules and peptides as ligands while uses proteins/nucleic acids as targets	Free	http://biophys.umontreal.ca/nrg/resources.html
144	LeDock	588	3.0786	Can be used for docking of ligands with protein target	Free	http://www.lephar.com/download.htm
145	MemDock	585	3.0764	Membrane protein docking algorithm	Free	http://bioinfo3d.cs.tau.ac.il/Memdock/php.php
146	TagDock	584	3.0757	Generates 3D models of oligomeric biomolecular complexes	Subscription	https://doi.org/10.1021/bi400158k
147	MDockPeP	580	3.0727	Docks the all-atom, flexible peptide onto the whole protein	Free	http://zougrouptoolkit.missouri.edu/mdockpep/
148	ASPDock	578	3.0712	Atomic Solvation Parameters (ASP) model which is used for calculating the	Subscription	http://biophy.hust.edu.cn/ASPDOCK.html

No.	Software	No. of citations to published studies	Score	Features	Accessibility	Website
				binding free energy of protein complexes		
149	PythDock	566	3.0621	Uses Python programming language with a simple scoring function and a population-based search engine	Subscription	https://doi.org/10.1007/s12272-011-0906-5
150	HDOCK	541	3.0425	Predicts the binding complexes between two molecules like proteins and nucleic acids by using a hybrid docking strategy	Free	http://hdock.phys.hust.edu.cn/
151	iGEMDOCK	536	3.0384	Can enrich the hit rate and provide biological insights by deriving the pharmacological interactions from screening compounds	Free	http://gemdock.life.nctu.edu.tw/dock/igemdock.php
152	DockRank	528	3.0319	Improves the scoring of docked poses, outperforms several energy-based scoring functions, and prioritizing ligands according to their affinity	Subscription	https://pubmed.ncbi.nlm.nih.gov/23873600/
153	ROSIE (Rosetta Online)	524	3.0286	Provides the 3D structure prediction and high-resolution design of natural and non-natural polymers	Free	https://rosie.rosettacommons.org/
154	ISE-Dock	492	3.0012	Eliminates values that consistently lead to the worst results, thus optimizing the search for docking poses	Subscription	https://pubmed.ncbi.nlm.nih.gov/18058908/
155	NRGsuite	490	2.9995	Gives access to powerful docking simulations that can be used in structure-guided drug design as well as an educational tool	Free	http://biophys.umontreal.ca/nrg/resources.html
156	evERdock	488	2.9977	Calculates binding free energy relaying on molecular dynamics (MD) simulations	Subscription	https://doi.org/10.1063/1.5019864
157	LeWater	474	2.9850	Filter docking poses and/or integrated into a scoring function to improve the accuracy of predicted binding affinity	Free	http://www.lephar.com/download.htm
158	LibDock	472	2.9832	Enable a safe and efficient loading and unloading while reducing downtime to a minimum	Free	https://www.bioz.com/result/libdock/product/Accelrys

No.	Software	No. of citations to published studies	Score	Features	Accessibility	Website
159	bhDock	463	2.9748	Uses two-step algorithm. First, low-resolution binding sites. Second, molecular dynamics	Free	DOI: 10.1002/jcc.21394
160	PLANTS	462	2.9739	PLANTS (Protein-Ligand ANT System), which is based on ant colony optimization	Subscription	https://doi.org/10.1021/ci800298z
161	VLifeDock	460	2.9720	Explores binding mode of ligand over a grid	Subscription	https://www.vlifesciences.com/products/Functional_products/VLifeDock.php
162	HiPCDock	432	2.9447	High-performance computing-based molecular docking scheme which is termed hipcdock	Subscription	https://pubmed.ncbi.nlm.nih.gov/19525200/
163	eSimDock	431	2.9437	Employs non-linear machine learning-based scoring functions to improve the accuracy of ligand ranking	Subscription	https://pubmed.ncbi.nlm.nih.gov/24171431/
164	DockingApp	400	2.9113	Explores the behavior of small molecules in the binding site of a target protein	Free	https://doi.org/10.1007/s10822-016-0006-1
165	DockDE	387	2.8970	Showed remarkable performance in terms of convergence speed and robustness regarding the found solution	Subscription	https://link.springer.com/article/10.1023/A:1008202821328
166	YUCCA	352	2.8558	A new algorithm for rigid protein–small-molecule docking	Subscription	https://doi.org/10.1002/cbdv.200590123
167	RPDOCK	348	2.8508	A docking procedure specific to RNA-protein complexes	Subscription	https://www.nature.com/articles/srep01887
168	ADAM	348	2.8508	Predicts a stable binding mode to target flexible ligand molecule macromolecule	Subscription	http://adam.vbi.vt.edu
169	QSAR-Co	346	2.8483	Setup classification-based QSAR models that allow mining the response data coming from multiple conditions	Free	https://sites.google.com/view/qsar-co
170	SymmRef	315	2.8076	Flexible backbone and side-chain refinement and re-scoring of symmetric rigid-body	Free	http://bioinfo3d.cs.tau.ac.il/SymmRef/php.php

No.	Software	No. of citations to published studies	Score	Features	Accessibility	Website
171	DockQ	310	2.8006	A continuous protein-protein docking model quality measure	Free	http://github.com/bjornwallner/DockQ/
172	HSYMDOCK	280	2.7564	Symmetric protein docking for oligomers with Cn or Dn symmetry from the structure or sequence of a subunit molecule	Free	http://huanglab.phys.hust.edu.cn/hsymdock/
173	MEGADOCK	256	2.7175	A structural bioinformatics software for FFT-grid-based protein-protein docking that takes advantage of CUDA architecture of NVIDIA graphics processing units (GPUs) and multiple computation nodes	Free	http://www.bi.cs.titech.ac.jp/megadock
174	Molegro	242	2.6931	Shown to outperform other docking programs with regard to identification of correct binding modes	Free	http://molexus.io/molegro-molecular-viewer/
175	LightDock	240	2.6895	Optimizes the generated docking poses towards those energetically more favourable at every simulation step	Free	https://github.com/brianjimenez/lightdock
176	MEGADOCK-GPU	232	2.6747	A fast, improved, protein-protein docking prediction by introducing a simple hydrophobic interaction model	Free	http://www.bi.cs.titech.ac.jp/megadock/gpu/
177	GetCleft	228	2.6672	Built-in and gives the possibility to refine and measure the volume of cavities	Free	http://biophys.umontreal.ca/nrg/resources.html
178	Snapdock	224	2.6595	Employs a Geometric Hashing-based structural alignment scheme to align the target proteins to the interfaces of non-redundant protein–protein interface libraries	Subscription	https://doi.org/10.1093/bioinformatics/btx233
179	ProQDock	219	2.6497	Uses support vector machines trained to predict the quality of protein docking models using features that can be calculated from the docking model itself	Free	http://bioinfo.ifm.liu.se/ProQDock
180	Ligin	193	2.5948	Uses surface complementarity approach for predicting the structure of ligand-receptor complex	Subscription	https://doi.org/10.1002/(SICI)1097-0134(1997)1+<210::AID-PROT28>3.0.CO;2-Q

No.	Software	No. of citations to published studies	Score	Features	Accessibility	Website
181	2dRNA	182	2.5693	Predicts RNA secondary structure	Free	http://biophy.hust.edu.cn/2dRNA/download
182	DockTrina	177	2.5572	A protein docking technique for demonstrating the 3D structures of nonsymmetrical triangular trimers	Free	https://team.inria.fr/nano-d/software/docktrina/
183	FITTED	175	2.5523	Can deal with both the flexibility of macromolecules (side chains and main chains) and the presence of bridging water molecules	Free	http://mgltools.scripps.edu/documentation/links/fitted
184	DockThor	158	2.5079	Performs pose prediction on redocking studies	Free	https://www.dockthor.lncc.br/
185	HYBRID	156	2.5024	The HYBRID program uses a modified version of FRED's algorithm that uses both ligand- and structure-based information to dock molecules	Subscription	https://www.eyesopen.com/oedocking-tk
186	PATIDOCK	154	2.4968	Relies solely on the 3D structure of the individual components and the experimentally derived Residual Dipolar Couplings (RDCs) for the complex	Subscription	https://doi.org/10.1021/ja100447p
187	TCRFlexDock	147	2.4766	Produces relatively accurate docking models (average interface RMSDs less than 2 A^{o} from bound)	Free	http://www.cbs.dtu.dk/services/TCRpMHCmodels/index.php
188	mtsslDock<br>mtsslSuite<br>mtsslTrilaterate<br>mtsslWizard	138	2.4491	Enables the user to dock two macromolecules based on a set of experimental PELDOR distances	Subscription	http://www.mtsslsuite.isb.ukbonn.de/
189	Schrödinger	133	2.4331	Delivers actual creditable and high achievement computational dependent technology to solution in life science and other applied science research	Free	https://www.schrodinger.com/freemaestro
190	SnugDock	95	2.2870	Predicts antibody-antigen complexes through optimization of antibody degrees of freedom relevant to binding	Subscription	https://www.rosettacommons.org/docs/latest/application_documentation/antibody/snugdock
191	ScoreDock	82	2.2231	Improves the docking accuracy and was shown to be reliable in docking quality assessment	Subscription	DOI: 10.1023/a:1011188704521

No.	Software	No. of citations to published studies	Score	Features	Accessibility	Website
192	DockAFM	70	2.1544	Establishes a link between topographic images from AFM and molecular dynamics of single proteins	Free	http://biodev.cea.fr/dockafm/
193	hint!	61	2.0946	An empirical molecular modelling system with new methods for de novo drug design and protein or nucleic acid structural analysis	Subscription	https://doi.org/10.1007/BF00135313
194	S3DB	44	1.9527	A database of manually curated target and ligand structures	Free	http://www.swissdock.ch/
195	SCIGRESS	41	1.9220	Computational chemistry integrated platform SCIGRESS	Subscription	https://doi.org/10.11546/cicsj.26.122
196	LeFrag	39	1.9003	Automatically decompose all small molecules in a library into fragments	Free	http://www.lephar.com/download.htm
197	iMOLSDOCK	36	1.8656	Uses mutually orthogonal Latin squares (MOLS) to sample the conformation and the docking pose of the ligand and also the flexible residues of the receptor protein	Subscription	https://doi.org/10.1016/j.jmgm.2017.03.008
198	molecularforecaster	35	1.8533	Used for drug discovery, metabolism prediction, combinatorial chemistry, and asymmetric catalyst design	Free	https://www.molecularforecaster.com/
199	MpSDock MpSDockZn	35	1.8533	A metalloprotein-specific docking program, which runs on a scheme similar to consensus scoring consisting	Subscription	http://www.lilab-ecust.cn/home/resource/14.html
200	Hammerhead	31	1.8006	Fast, fully automated docking of flexible ligands to protein binding sites	Subscription	doi:10.1016/s1074-5521(96)90093-9
201	UDock	31	1.8006	Interactive protein docking system, intended for both naive and experienced users	Free	http://udock.fr
202	MEGADOCK-Web	29	1.7717	A database of protein-protein interactions (ppis) predicted by an FFT-grid-based protein-protein docking software, MEGADOCK	Free	http://www.bi.cs.titech.ac.jp/megadock-web/

No.	Software	No. of citations to published studies	Score	Features	Accessibility	Website
203	DockScore	18	1.5645	Can be utilized to rank protein-protein docked poses	Free	http://caps.ncbs.res.in/dockscore/
204	DockAnalyse	18	1.5645	Analysis of protein-protein interactions	Free	http://bioinf.uab.es/rker/DockAnalyse/DockAnalyse.zip
205	mApfDock	13	1.4232	The docking of candidates to atomic property fields derived by co-crystallized ligands	Subscription	https://doi.org/10.4155/fmc.14.113
206	mPockDock	13	1.4232	Reduces the rate of false-negatives in activity prediction by docking flexible 3D models of compounds include multi-conformational docking	Subscription	https://doi.org/10.4155/fmc.14.113
207	FlexGAsDock	13	1.4232	Based on the hierarchical docking strategy	Subscription	http://en.cnki.com.cn/Article_en/CJFDTOTAL-DLLG200802024.htm
208	ELMDOCK ELMPATIDOCK	12	1.3884	Relies solely on the 3D structure of the individual components and the overall rotational diffusion tensor of the complex	Subscription	https://onlinelibrary.wiley.com/doi/abs/10.1002/prot.23053
209	BDT-AutoDock	3	0.7864	Using receptor flexibility during docking using a large library of ligands onto one or more receptors without defining one a priori ligand-binding site on them	Free	http://www.quimica.urv.cat/~pujadas/BDT/
210	Discovery Studio	0	0.0000	An interactive package with a broad variety of features for modelling and simulation	Subscription	https://www.bu.edu/tech/support/research/software-and-programming/software-and-applications/rcs-software-packages/discovery-studio/
211	3dRNA-RFSP	0	0.0000	Constructs a statistical potential for 3D RNA structure evaluation by using an iterative method that circumvents the reference state problem	Free	http://biophy.hust.edu.cn/3dRNA-RFSP.html
